# Population attributable fractions of fatty liver disease for type 2 diabetes Mellitus

**DOI:** 10.1186/s12902-023-01433-z

**Published:** 2023-09-19

**Authors:** Jingyuan Xu, Longyun Wu, Jiang Deng, Longbao Yang, Yatao Wang, Hongyang Tian, Yajun Ji, Qiaoyun Xia, Zhiyi Han, Yan Cheng, Xiaolan Lu

**Affiliations:** 1 Department of Gastroenterology, The Second Affiliated Hospital of Xi’an Jiaotong University, Xi’an, 710004 China; 2 Department of Gastroenterology, The Affiliated Suzhou Hospital of Nanjing Medical University, 215001, 16 Baita West Road, Suzhou, China; 3 Department of Gastroenterology, Shanghai Pudong Hospital of Fudan University, Shanghai, 201399 China; 4 Karamay Central Hospital of Xinjiang, Karamay, 834099 China

**Keywords:** Fatty liver disease, Type 2 diabetes mellitus, Population attributable fraction, Cohort studies, Metabolic diseases

## Abstract

**Purpose:**

To determine the population attributable fraction (PAF) of fatty liver disease (FLD) for type 2 diabetes mellitus (T2DM) and compare it to the PAFs of other metabolic abnormalities.

**Methods:**

We conducted a 10-year retrospective cohort study of 33,346 individuals in Karamay Central Hospital of Xinjiang. Individuals were followed up for T2DM occurrence based on FBS. The PAFs of FLD were calculated generally and respectively in different sex and age groups. A comparison of the PAF of FLD and that of other metabolic abnormalities, as well as the PAFs of FLD in different groups classified based on age and sex, was performed using Cox regression.

**Results:**

During an average follow-up period of 3.71 years, 1486 T2DM were diagnosed. The incidence density of T2DM was 1.2/100 person-years, and cumulative incidence rate was 4456.31/100,000 person-years. Partial PAF (PAF_p_) of FLD in the entire population was 23.11%. In the male population, PAF_p_ was higher at 30–40 years old. In the female population, it was higher when age ≥ 60 years old. In multivariable Cox regression model, FLD, male sex, age ≥ 45 years old, overweight, hypertriglyceridaemia, and systolic hypertension were independent risk factors for T2DM, with corresponding PAF_p_ of 25.00%, 24.99%, 36.47%, 24.96%, 5.71%, and 6.76%, respectively. Age ≥ 45 years old showed the highest PAFp and adjusted hazard ratio, followed by FLD.

**Conclusions:**

FLD contributes more to T2DM incidence than other metabolic disorders. Particular attention should be given to male populations of 30–40 and female populations above 60 for FLD prevention and treatment.

## Introduction

Type 2 diabetes mellitus (T2DM) poses a heavy public health burden. In China, a large number of individuals were affected by T2DM, which not only leads to foot disease, neuropathic, nephropathic and retinopathic damage, but also induces cardiovascular and cerebrovascular events, directing caused the increased mortality by T2DM-related disease [1]. With the epidemics of overweight and obesity, urbanization, and the aging trend, there is a constant increment of the prevalence of T2DM annually, and it has risen steadily over the last 30 years [1, 2]. In 1980, the prevalence of T2DM in China was only 0.67%. According to the 2008 China national diabetes and metabolic disorders study, the estimated T2DM in 20-year-old patients was over 92.4 million, with a prevalence of 9.7%. In adults, the prevalence of T2DM was 9.7% in 2010 and had reached 11.2% in 2017 [1]. Despite the discovery of multiple potential therapeutic targets, the effective medicine against T2DM is still largely lacking [1, 2]. Considering its uprising trend and grievous consequences, lifestyle intervention for high-risk populations is often employed to help to reduce the risk of T2DM [1]. Thus, identifying the high-risk populations for T2DM is momentous.

Fatty liver disease (FLD) is the most common hepatopathy worldwide with an overall prevalence rate of 40% and almost a fifth was lean [3–5]. It encompasses a range of conditions, including simple fatty liver, steatohepatitis, fibrosis, and cirrhosis [6, 7]. FLD is commonly associated with T2DM and other metabolic disorders, and there are about a third to two thirds of T2DM patients have FLD [3]. Numerous studies have proven that FLD, including steatohepatitis, is an independent risk factor for T2DM, and it is considered a modifiable factor in T2DM risk management and prevention [8–11]. In addition, studies suggested a bidirectional causal association between FLD and T2DM, and this is reflect in the fact that not only do FLD increase the incidence of T2DM, but T2DM can also promote the progression of FLD. [1, 3, 7]. Thus, there should be a high index of suspicion for T2DM in patients with FLD. T2DM is often accompanied by one or more metabolic abnormalities, such as hypertension, dyslipidaemia, and obesity, and these metabolic abnormalities enhance the development and progression of T2DM [1]. Many studies have proven that FLD and other metabolic disorders increase the risk of T2DM [12–15], but the population attributable fraction (PAF) of FLD for T2DM is rarely reported. Owing to the insulin resistance and hyperinsulinemia, which are typical pathological features of T2DM, T2DM escalate adverse cardiovascular outcomes. Significantly, FLD and steatohepatitis not only increase the prevalence of T2DM, but also considerably contributes to insulin resistance and hyperinsulinemia [1, 9, 12]. PAF is a valuable statistic in quantifying the burden of a specific disease which was assessed by the pooled relative risk (RR) [16, 17]. By examining the risk reduction of diseases in a specific population after eliminating one or several risk factors alone or adjusting the influence of other risk factors, it provides an estimate of the disease risk that can be attributed to certain risk factors in a given population, and was served as an useful tool for the prevention and research of multiple disease aetiologies [16, 17]. In this study, we aimed to investigate the PAF of FLD for T2DM and compare it to the PAFs of other metabolic syndromes, as well as to assess the PAFs of FLD in different sex and age groups to identify specific high-risk population groups for T2DM.

## Materials and methods

### Population

**Fig. 1:**
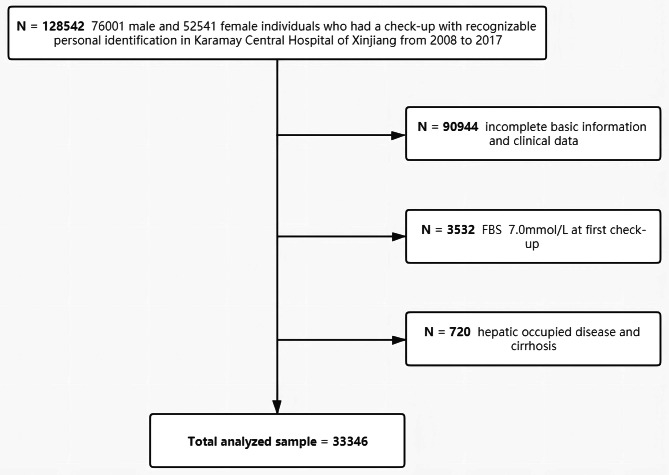
Participant flowchart for analysis in the retrospective cohort study. Incomplete basic information and clinical data for analysis: sex, age, body mass index (BMI), systolic blood pressure (SBP), diastolic blood pressure (DBP), triglyceride (TG), total cholesterol (TC), high-density lipoprotein cholesterol (HDL), low-density lipoprotein cholesterol (LDL), aspartate aminotransferase (AST), alanine aminotransferase (ALT), fasting blood sugar (FBS), and abdominal ultrasonography

We conducted a retrospective cohort study in non-T2DM individuals. Hazard ratios (HRs) and PAFs of FLD were estimated generally and respectively in different sex and age groups. The HRs and PAFs for T2DM of FLD and other metabolic disorders, as well as those for T2DM of FLD in different groups based on age and sex, were analysed and compared. For this study, 76,001 men and 52,541 women were recruited from individuals who had a check-up with recognisable personal identification in Karamay Central Hospital of Xinjiang from 2008 to 2017. Exclusion criteria were as follows (Fig. 1): (1) incomplete basic information and clinical data (90,944); (2) fasting blood sugar (FBS) ≥ 7.0 mmol/L at the initial or previous check-up (3532); and (3) hepatic occupied disease and cirrhosis (720). Finally, a total of 33,346 individuals were included in the study.

### Baseline data and examinations

Clinical data and laboratory examinations included sex, age, nationality, weight, height, abdominal ultrasound, FBS, systolic blood pressure (SBP), diastolic blood pressure (DBP), aspartate aminotransferase (AST), alanine aminotransferase (ALT), triglyceride (TG), total cholesterol (TC), high-density lipoprotein cholesterol (HDL), and low-density lipoprotein cholesterol (LDL).

### FLD ascertainment

Fatty liver disease (FLD) was defined by ultrasound [3, 26]: parenchymal brightness, liver to kidney contrast, deep beam attenuation, bright vessel walls, and gallbladder wall definition.

### FBS follow-up and T2DM ascertainment

Individuals were followed up for T2DM as a terminal event. The follow-up time started from October 2008 and was censored past April 2017. Individuals had at least one FBS test each year to ascertain T2DM onset, which was diagnosed when FBS was ≥ 7.0 mmol/L. Individuals with no FBS results for 24 consecutive months were considered lost to follow-up.

### PAFs for T2DM

PAF was calculated as *p* (RR − 1) / 1 + *p* (RR − 1), *p* is the prevalence of T2DM. The adjusted PAFs of FLD for T2DM were estimated as described [17]. The individuals were divided into FLD and non-FLD groups, and the T2DM incidence was analysed. Other clinical data were divided as follows [18–20]: age ≥ 45 years, overweight [BMI (body mass index) ≥ 24 kg/m^2^], systolic hypertension (SBP ≥ 140 mmHg), diastolic hypertension (DBP ≥ 90 mmHg), hypertriglyceridaemia (TG > 1.7 mmol/L), hypercholesterolaemia (TC > 5.8 mmol/L), low HDL (HDL < 1.8 mmol/L), and high LDL (LDL > 3.3 mmol/L). We estimated the crude PAFs and crude HRs in a cohort study of FLD. To further analyse the crude PAFs (PAF_c_) and HRs of FLD in the different sex and age groups and identify specific high-risk population groups for T2DM, we divided the total population into < 45 years old, 45–59 years old, and ≥ 60 years old groups. We stratified data by sex and analysed the PAF_c_ and HRs of FLD between groups classified as age ≥ 45 years old and those based on sex. We adjusted for sex, age, BMI, SBP, DBP, HDL, LDL, TG, and TG to obtain partial PAFs (PAF_p_) and adjusted HRs, after which the PAF_p_ and adjusted HRs of FLD were compared with those of other metabolic disorders in another multivariable Cox regression model.

### Statistical analysis

All statistical analyses were conducted using IBM SPSS Statistics 26.0 (SPSS, Inc., Chicago, Illinois), STATA 15.0 and CRAN-R 3.6.2. All statistical analyses used a two-tailed test, and P < 0.05 was considered to indicate statistical significance. We set T2DM as the outcome and endpoint. Kaplan–Meier curve differences were assessed by log-rank test. The Cox regression model was used to identify the independent risk factors and calculate HRs. PAFs were used to estimate the incidence rate of the whole population, which was attributed to the exposure of the risk factors. We estimated the crude HRs and their corresponding PAF_c_, as well as adjusted HRs and their corresponding partial PAF_p_. We calculated the 95% confidence interval for HRs and PAFs.

## Results

A total of 33,346 individuals were followed up for 123694.84 person-years. The average follow-up period was 3.71 years, and 1486 individuals (4.46%) with T2DM were identified. The incidence density of T2DM was 1.2/100 person-years, and the cumulative incidence rate was 4456.31/100 thousand person. Demographic and clinical characteristics between the FLD and non-FLD groups are presented in Table 1. Individuals in the FLD group were more likely to have higher age, BMI, TC, TG, SBP, DBP, and LDL and lower HDL than those in the non-FLD group.

**Fig. 2:**
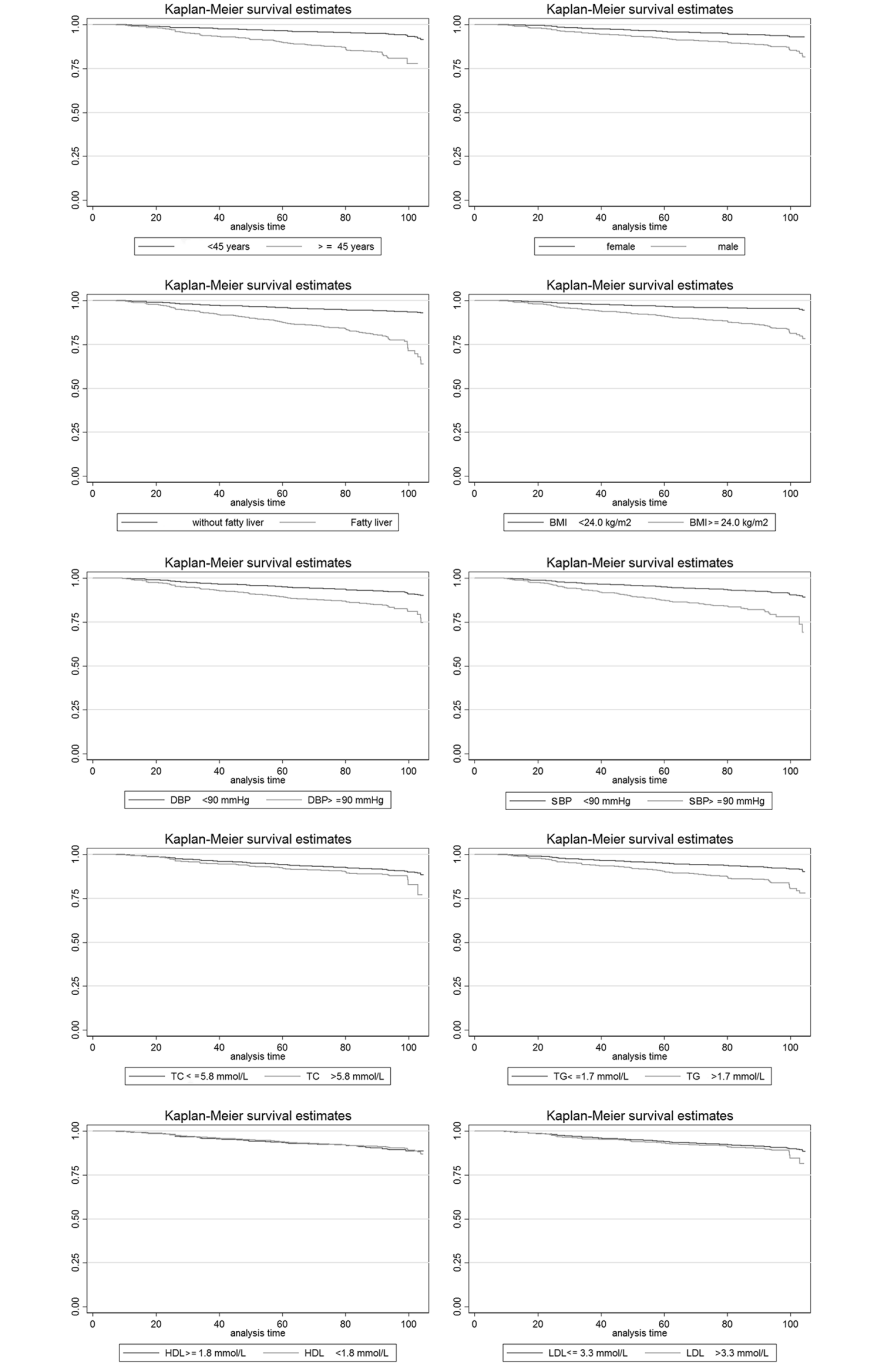
Kaplan-Meier analysis for T2DM incidence. For participants with FLD, age ≥ 45 years, BMI ≥ 24 kg/m2, SBP ≥ 140 mmHg, DBP ≥ 90 mmHg, TG > 1.7 mmol/L, TC > 5.8 mmol/L, HDL < 1.8 mmol/L, LDL > 3.3 mmol/L and different gender, the cumulative survival rate for T2DM incidence was analyzed per month

**Table 1:** Baseline information between FLD group and non-FLD group

		non-FLD group (n = 24,435)		FLD group (n = 8911)		Pearson Chi-Square	
		Number	Percentage	Number	Percentage	χ value	*P* value
Sex	male	12,323	50.4%	6667	74.8%	1583.803	< 0.01
	female	12,112	49.6%	2244	25.2%		
Age	< 45 years	15,965	65.3%	4738	53.2%	410.602	< 0.01
	≥ 45 years	8470	34.7%	4173	46.8%		
BMI	< 24.0 kg/m2	15,437	63.2%	1063	11.9%	6860.149	< 0.01
	≥ 24.0 kg/m2	8998	36.8%	7848	88.1%		
TC	≤ 5.8 mmol/L	20,979	85.9%	6811	76.4%	417.528	< 0.01
	> 5.8 mmol/L	3456	14.1%	2100	23.6%		
TG	≤ 1.7 mmol/L	20,343	83.3%	4187	47.0%	4415.994	< 0.01
	> 1.7 mmol/L	4092	16.7%	4724	53.0%		
HDL	≥ 1.8 mmol/L	4997	20.5%	1565	17.6%	34.447	< 0.01
	< 1.8 mmol/L	19,438	79.5%	7346	82.4%		
LDL	≤ 3.3 mmol/L	19,121	78.3%	6462	72.5%	120.262	< 0.01
	> 3.3 mmol/L	5314	21.7%	2449	27.5%		
SBP	< 140 mmHg	21,628	88.5%	6503	73.0%	1194.468	< 0.01
	≥ 140 mmHg	2807	11.5%	2408	27.0%		
DBP	< 90 mmHg	20,621	84.4%	5583	62.7%	1833.361	< 0.01
	≥ 90 mmHg	3814	15.6%	3328	37.3%		

**Fig. 3:**
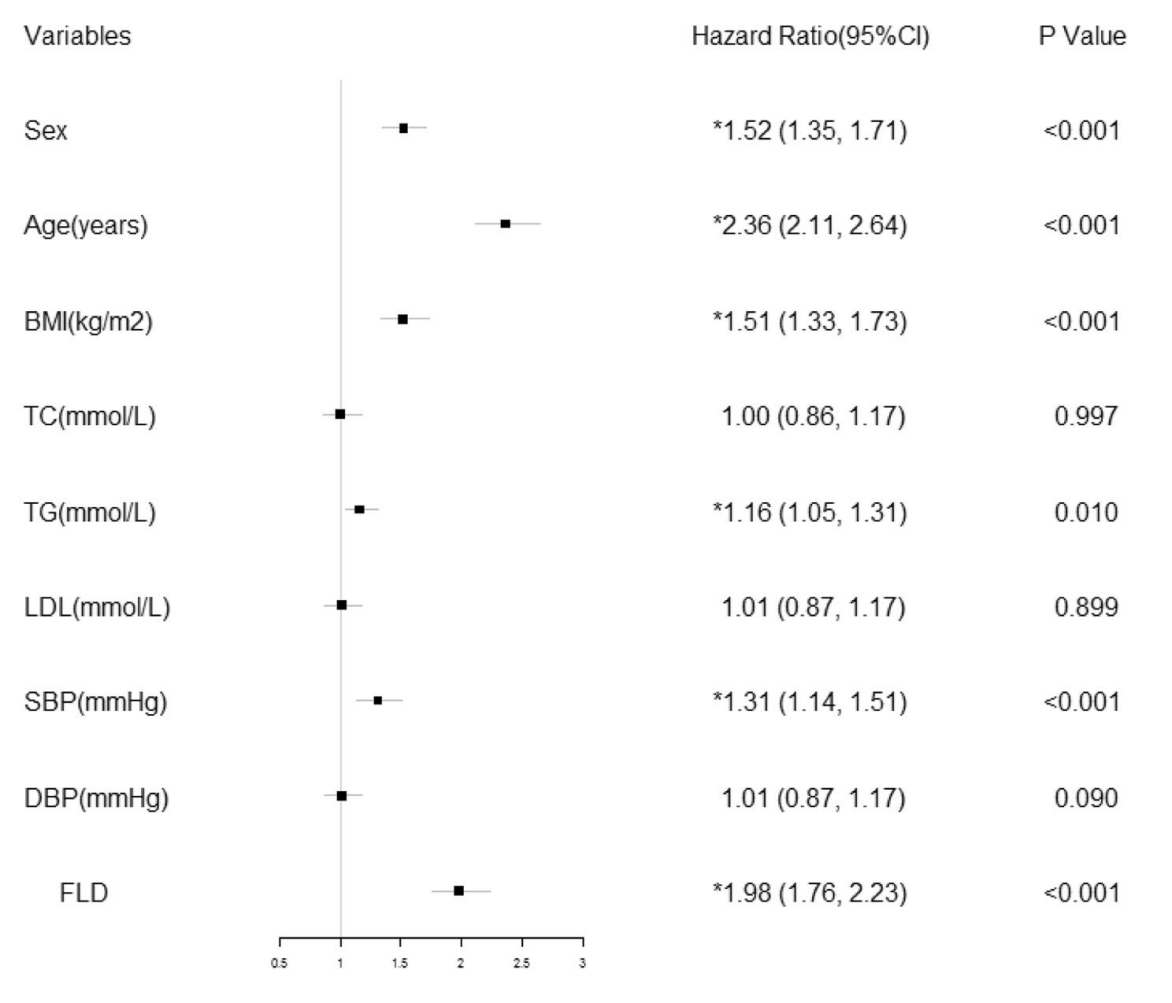
HRs in multivariable Cox regression model. HRs in Cox regression model with adjusted factors. Sex, age, BMI, TC, TG, LDL, SBP, DBP, and NAFLD variables refer to the sex group, age group, BMI group, TC group, TG group, LDL group, SBP group, DBP group, and FLD or non-FLD group, respectively

In Kaplan–Meier analysis (Fig. 2), we found that the cumulative survival rate of T2DM was higher in the non-FLD group than FLD group (*P* < 0.01) (Table 2). The PAF_c_ of FLD for T2DM was 34.77% (31.35%, 38.02%), after adjusting the influence of BMI, TC, TG, HDL, LDL, SBP, and DBP. The PAF_p_ was 23.11% (21.83%, 47.99%), which indicates that 343 (range 324–713) T2DM cases were caused by FLD during the follow-up time in the population (Table 2). The HRs and PAFs of FLD in different sex and age groups are shown in Tables 2 and 3; Fig. 3.

**Table 2:** PAF in different sex and age group of FLD for T2DM

	Total		Male		Female	
	PAF_c_ (%)	PAF_p_^#^(%)	PAF_c_ (%)	PAF_p_^#^(%)	PAF_c_ (%)	PAF_p_^#^(%)
**Total**	^*^34.77 (31.35, 38.02)	^*^23.11 (18.47, 27.49)	^*^30.43 (25.91, 34.68)	^*^21.16 (15.43, 26.50)	^*^34.37 (28.53, 39.74)	^*^25.36 (17.75, 32.27)
**age group**						
< **45**	^*^37.47 (31.98, 42.52)	^*^23.31 (15.59, 30.32)	^*^38.02 (30.49, 44.73)	^*^23.12 (12.71, 32.29)	^*^23.46 (15.12, 30.98)	^*^18.48 (0.90, 27.01)
(1) < 30	^*^36.24 (21.83, 47.99)	^*^22.72 (3.00, 38.44)	^*^35.78 (18.66, 49.29)	17.41 (-7.70, 36.67)	22.18 (-7.64, 43.73)	24.34 (-4.40, 45.17)
(2) 30 ~ < 35	^*^47.86 (31.93, 60.05)	^*^39.53 (17.86, 55.49)	^*^44.67 (23.93, 59.75)	^*^36.07 (8.86, 55.16)	^*^40.07 (5.74, 61.90)	36.90 (-1.48, 60.76)
(3) 35 ~ < 40	^*^35.13 (24.86, 44.00)	^*^23.15 (8.45, 35.49)	^*^40.52 (25.40, 52.59)	^*^32.41 (13.09, 47.44)	6.97 (-3.61, 16.47)	-2.48 (-19.47, 12.08)
(4) 40 ~ < 45	^*^35.15 (26.08, 43.11)	^*^18.96 (6.83, 29.51)	^*^30.16 (16.10, 41.86)	13.64 (4.38, 28.55)	^*^28.86 (15.61, 40.03)	^*^23.26 (8.73, 35.47)
**45 ~ < 60**	^*^30.57 (24.77, 35.93)	^*^22.47 (15.08, 29.22)	^*^27.84 (20.28, 34.68)	^*^23.82 (14.96, 31.77)	^*^28.53 (18.35, 37.43)	^*^18.98 (5.57, 30.48)
(1) 45 ~ < 50	^*^32.10 ( 21.95, 40.92)	^*^21.05 (7.42, 32.69)	^*^29.01 (14.55, 41.03)	^*^21.77 (4.32, 36.03)	^*^22.30 (6.03, 35.75)	13.41 (-8.27, 30.76)
(2) 50 ~ < 55	^*^30.25 (19.73, 39.40)	^*^24.35 (11.31, 35.47)	^*^24.85 (10.77, 36.72)	^*^21.96 (5.60, 35.48)	^*^34.65 (16.55, 48.82)	^*^29.09 (7.44, 45.68)
(3) 55 ~ < 60	^*^29.47 (19.14, 38.48)	^*^24.28 (11.93, 34.90)	^*^32.63 (20.97, 42.57)	^*^29.62 (16.36, 40.78)	22.25 (-1.04, 40.17)	12.61 (-20.27, 36.51)
**≥ 60**	^*^27.12 (19.10, 34.34)	^*^21.70 (12.09, 30.25)	^*^20.68 (11.90, 28.59)	^*^15.97 (5.58, 25.21)	^*^49.59 (31.38, 62.97)	^*^44.01 (21.35, 60.14)
(1) 60 ~ < 65	^*^34.55 (19.27, 46.94)	^*^31.26 (14.14, 44.97)	^*^26.75 (8.44, 41.40)	^*^23.20 (2.12, 39.73)	^*^53.05 (22.58, 71.53)	^*^52.43 (20.94, 71.38)
(2) ≥ 65	^*^23.29 (13.79, 31.75)	^*^17.94 (6.46, 28.01)	^*^18.19 (8.21, 27.08)	^*^14.21 (2.39, 24.61)	^*^46.35 (20.78, 63.67)	35.14 (-3.50, 59.36)

^*^P < 0.05 and 95% CI do not include zero of PAF_c_ and PAF_p_; ^#^ Adjusted for BMI, TC, TG, HDL, LDL, SBP, DBP

**Table 3:** HR in different sex and age group of FLD for T2DM

	Total		Male		Female	
	Crude HR	Adjusted HR^#^	Crude HR	Adjusted HR^#^	Crude HR	Adjusted HR^#^
**Total**	^*^3.20 (2.88, 3.54)	^*^1.84 (1.62, 2.09)	^*^2.33 (2.07, 2.63)	^*^1.66 (1.44, 1.91)	^*^4.87 (3.98, 5.97)	^*^2.42 (1.85, 3.16)
**age** *year*						
**18 ~ < 45**	^*^3.95 (3.33, 4.68)	^*^1.87 (1.51, 2.31)	^*^2.92 (2.30, 3.56)	^*^1.67 (1.32, 2.10)	^*^5.26 (3.67, 7.55)	^*^2.76 (1.73, 4.41)
(1) 18 ~ < 30	^*^4.02 (2.63, 6.15)	^*^1.89 (1.12, 3.20)	^*^3.20 (2.03, 5.04)	1.50 (0.88, 2.57)	^*^8.86 (2.46, 31.84)	^*^37.87 (10.93, 131.14)
(2) 30 ~ < 35	6.27 (3.87, 10.15)	^*^3.27 (1.66, 6.44)	^*^3.85 (2.25, 6.60)	^*^2.49 (1.27, 4.88)	^*^15.39 (5.18, 45.76)	^*^7.19 (1.48, 34.91)
(3) 35 ~ < 40	^*^3.93 (2.86, 5.39)	^*^1.97 (1.28, 3.01)	^*^3.03 (2.07, 4.42)	^*^2.15 (1.36, 3.41)	2.50 (0.98, 6.34)	0.82 (0.24, 2.83)
(4) 40 ~ < 45	^*^3.24 (2.49, 4.21)	^*^1.59 (1.19, 2.13)	^*^2.10 (1.52, 2.89)	1.31 (0.94, 1.83)	^*^4.62 (2.89, 7.37)	^*^2.71 (1.57, 4.68)
**45 ~ < 60**	^*^2.35 (2.02, 2.74)	^*^1.73 (1.44, 2.08)	^*^1.99 (1.66, 2.38)	^*^1.74 (1.41, 2.15)	^*^2.61 (1.95, 3.50)	^*^1.70 (1.18, 2.44)
(1) 45 ~ < 50	^*^2.47 (1.90, 3.20)	^*^1.64 (1.19, 2.25)	^*^1.93 (1.41, 2.63)	^*^1.57 (1.10, 2.22)	^*^2.50 (1.49, 4.21)	1.57 (0.81, 3.04)
(2) 50 ~ < 55	^*^2.28 (1.75, 2.98)	^*^1.83 (1.32, 2.52)	^*^1.84 (1.33, 2.53)	^*^1.67 (1.15, 2.43)	^*^2.98 (1.85, 4.80)	^*^2.26 (1.27, 4.03)
(3) 55 ~ < 60	^*^2.32 (1.77, 3.03)	^*^1.88 (1.37, 2.58)	^*^2.62 (1.92, 3.57)	^*^2.28 (1.59, 3.27)	^*^1.83 (1.06, 3.15)	1.35 (0.68, 2.67)
**≥ 60**	^*^2.51 (1.98, 3.18)	^*^1.93 (1.45, 2.56)	^*^2.17 (1.64, 2.87)	^*^1.71 (1.238, 2.39)	^*^4.26 (2.62, 6.94)	^*^3.12 (1.71, 5.69)
(1) 60 ~ < 65	^*^2.74 (1.85, 4.06)	^*^2.35 (1.48, 3.72)	^*^2.23 (1.39, 3.55)	^*^1.91 (1.10, 3.34)	^*^4.61 (2.18, 9.77)	^*^4.42 (1.90, 10.28)
(2) ≥ 65	^*^2.41 (1.78, 3.27)	^*^1.82 (1.27, 2.62)	^*^2.22 (1.55, 3.18)	^*^1.75 (1.14, 2.70)	^*^3.87 (2.04, 7.34)	2.28 (0.98, 5.33)

^*^P < 0.05 and 95% CI do not include one of crude and adjusted HR; ^#^ Adjusted for BMI, TC, TG, HDL, LDL, SBP, DBP

In the whole population, the PAF_p_ of FLD was 23.31% in individuals under 45 years old, 22.47% in 45–59 years old individuals, and 21.70% in individuals above 60 years old. In the male population, the PAF_p_ was 23.12% in individuals under 45 years old, 23.82% in 45–59 years old individuals, and 15.97% in individuals above 60 years old. In the female population, the PAF_p_ was 18.48%, 18.98%, and 44.01%, respectively. The three age groups were further subdivided into nine groups. In the entire population, PAF_p_ (range 17.94–39.53%) was statistically significant in each age group. In the male population, the highest PAF_p_ was found in the 30- to 40-year age group (32.41% and 36.07%), and PAF_p_ was statistically significant in seven of the nine groups. In the female population, PAF_p_ showed statistical significance only in three groups: 40–45 (23.26%), 50–55 (29.09%), and 60–65 (52.43%).

The PAFs and HRs of FLD were compared with those of other metabolic disorders (Tables 4 and 5). Statistically significant adjusted HRs were obtained in age ≥ 45 years old, FLD, male sex, overweight, systolic hypertension, and hypertriglyceridaemia groups. A significant PAF_p_ was obtained for age ≥ 45 years old, overweight, male sex, FLD, systolic hypertension, and hypertriglyceridaemia groups. Age ≥ 45 years old had the highest adjusted HRs and PAF_p_, followed by FLD.

**Table 4:** Log-rank test and Cox regression model of sex, age, FLD and other metabolic disorders for T2DM

	χ^2^ value^a^	*P* value^b^	HR^c^	*P* value^d^	HR^e^	*P* value^f^
FLD	554.96	< 0.01	1.98(1.76,2.23)	< 0.01	NA	NA
male sex	187.81	< 0.01	1.52(1.35,1.71)	< 0.01	1.55(1.37,1.75)	< 0.01
middle-aged and elderly	420.28	< 0.01	2.36(2.11,2.64)	< 0.01	2.46(2.20,2.75)	< 0.01
overweight	365.81	< 0.01	1.51(1.33,1.73)	< 0.01	1.79(1.58,2.03)	< 0.01
hypercholesterolemia	25.63	< 0.01	1.00(0.86,1.17)	1.00	0.99(0.84,1.16)	0.88
hypertriglyceridemia	190.26	< 0.01	1.16(1.05,1.31)	0.01	1.27(1.13,1.42)	< 0.01
low HDL	1.13	0.29	/	/	/	/
high LDL	7.42	0.01	1.01(0.87,1.17)	0.90	1.01(0.87,1.16)	0.94
systolic hypertension	266.15	< 0.01	1.31(1.14,1.51)	< 0.01	1.34(1.17,1.55)	< 0.01
diastolic hypertension	229.78	< 0.01	1.12(0.98,1.28)	0.09	1.15(1.00,1.31)	0.05

^a^ χ^2^ value of log-rank test; ^b^*P* value of log-rank test; ^c^ HRs in Cox regression model with adjusted factors: FLD, male sex, middle-aged and elderly, overweight, hypercholesterolemia, hypertriglyceridemia, low HDL, systolic hypertension, diastolic hypertension; ^d^*P* value of HR^c^; ^e^ HR in Cox regression model with adjusted factors: male sex, age group, BMI group, TC group, TG group, LDL group, SBP group, DBP group; ^f^*P* value of HR ^e^

**Table 5:** Analysis of PAF of sex, age, FLD and other metabolic disorders for T2DM incidence

Factors	Crude HR(95%CI)	Adjusted HR(95%CI)	PAF_c_(95%CI)	PAF_p_(95%CI)
Sex female (reference) male	2.19(1.95, 2.45)	^*^1.52 (1.35, 1.71)	39.65% (34.41%, 44.48%)	^#^24.99% (18.04%, 31.35%)
Age(years) < 45 (reference) ≥45 years	2.92 (2.62, 3.25)	^*^2.36 (2.11, 2.64)	41.59% (37.56%, 45.37%)	^#^36.47% (31.96%, 40.68%)
BMI(kg/m^2^) < 24.0(reference) ≥24.0	2.93(2.61, 3.28)	^*^1.51 (1.33, 1.73)	48.37% (43.81%, 52.55)	^#^24.96% (17.32%, 31.89%)
TC(mmol/L) ≤5.8(reference) > 5.8	1.29(1.14, 1.46)	1.00 (0.86, 1.17)	5.02% (2.40%, 07.57%)	1.00% (-3.61%, 3.50%)
TG(mmol/L) ≤1.7(reference) > 1.7	2.04 (1.84, 2.27)	^*^1.16 (1.05, 1.31)	20.65% (17.29%, 23.88%)	^#^5.71% (1.26%, 9.96%)
LDL(mmol/L) ≤3.3(reference) > 3.3	1.17 (1.05, 1.31)	1.01 (0.87, 1.17)	3.96% (0.96%, 6.87%)	0.25% (-3.72%, 4.07%)
SBP(mmHg) < 140(reference) ≥140	2.48 (2.22, 2.78)	^*^1.31 (1.14, 1.51)	17.15% (14.46%, 19.75%)	^#^6.76% (3.10%, 10.28%)
DBP(mmHg) < 90(reference) ≥90	2.22 (1.99, 2.46)	1.01 (0.87, 1.17)	20.12% (17.01%, 23.12%)	4.00% (-0.80%, 8.57%)
FLD No (reference) Yes	3.20 (2.88, 3.54)	^*^1.98 (1.76, 2.23)	34.77% (31.35%, 38.02%)	^#^25.00% (20.73%, 29.04%)

^*^P < 0.05 and 95% CI do not include one of adjusted HR; ^#^*P* < 0.05 and 95% CI do not include zero of PAF_p_

## Discussion

In this retrospective cohort study, PAFs of FLD were estimated to determine the contribution of FLD to T2DM incidence. Sex, age, BMI, SBP, DBP, HDL, LDL, TC, and TG were adjusted in the multivariable Cox regression model to further eliminate their influence on T2DM incidence. The adjusted PAF_p_ was 23.11% in the overall population, 21.16% in the male population, and 25.36% in the female population, all of which showed statistical significance. Although the prevalence of FLD in male population was much higher than that in female population, which were consistent with most of the recent data[3, 26], our results indicated that FLD in female population showed a greater impact on T2DM incidence. Thus, the prevention and treatment for FLD in female population are of great importance and should not be unheeded. It was reported that the prevalence of FLD appeared to increase with age, and male gender was considered a risk factor of FLD [3]. In our study, the enrollees of different gender were stratified into subgroups based on age, and in the male population, the PAF_p_ was higher in < 45 years old and 45–59 years old groups than in ≥ 60 years old group, and it was higher in 45–59 years old group than in < 45 years old group. Accordingly, it is important to recognize that this particular group in male population with FLD have an especially high incidence of T2DM. In the female population, the PAF_p_ in ≥ 60 years old group was more than 2.3 times higher than that in ≥ 45 years old, hinted an enhanced insulin resistance influenced by hormone level in postmenopausal women with FLD [21–23], and it could be argued that there should be systematic screening and treatment for FLD among female individuals over 60 years old. Even though the PAF_p_ in the female population was higher; the PAF_p_s of only three groups showed statistical significance. In the male population, the PAF_p_s of seven of the nine age groups showed statistical significance. This finding may not be related to a large sufficient sample, but it provided some evidence that FLD had a more general influence on T2DM incidence in the male population. The PAF_p_ in the different age groups also differed between the male and female populations. FLD contributed the most to T2DM incidence in the 30- to 40-year age group in the male population. Since individuals ≥ 45 years old also had a high risk for T2DM in our study, this result seems reasonable. In the female population, FLD seemed to contribute more to T2DM incidence in individuals aged > 40 years. We can speculate that this may be caused by menopause because premenopausal hormonal levels prevent women from developing T2DM [21–23].

In another multivariable analysis that compared the HRs and PAFs of FLD with those of other metabolic disorders, the HR and PAF estimates highlighted the magnitude of age, sex, and metabolic abnormalities at the onset of diabetes. In addition to FLD, age (≥ 45 years old), male sex, overweight, hypertriglyceridaemia, and systolic hypertension were also identified to be independent factors influencing the onset of T2DM. In our study, FLD was ranked as the most common metabolic cause of T2DM, and the above results provided a data-supported approach to the primary prevention of T2DM, including the prevention and treatment of FLD, overweight, triglycerides, and systolic hypertension, which were consistent with previous studies [1, 8–15]. Age (≥ 45 years old) was the most important factor in our study, contributing to 36.47% of cases of onset of T2DM, followed by FLD in 25.00% of cases. Male sex is also associated with a higher onset of T2DM [1, 24], which is consistent with our conclusion. The PAF_p_ of the male sex was only 0.01% lower than that of FLD in our study. The PAF_p_ in the overweight group is 0.04% lower than that of FLD in this multivariable analysis, whereas PAFs of hypertriglyceridaemia and systolic hypertension were much lower. Age ≥ 45 years old showed the highest adjusted HRs and PAF_p_; however, physiological dysfunction associated with increased age could be difficult to modify by medical or lifestyle interventions. Male sex was the third leading cause of T2DM in our study; however, it is not a feasible factor for modification to reduce disease risk. Therefore, regulation of weight, FLD, systolic hypertension, and hypertriglyceridaemia is more practical for the reduction of disease risk. Compared to systolic hypertension and hypertriglyceridaemia, overweight and FLD contribute far more to T2DM. Moreover, men ≥ 45 years old should attach more importance to cultivating a healthy lifestyle and controlling metabolic abnormalities, such as FLD, overweight, hypertriglyceridaemia, and systolic hypertension, to prevent T2DM occurrence.

FLD, the most important modifiable metabolic factor in our study, often exists concurrently with insulin resistance [25]. Early intervention and treatment of FLD not only prevent it from developing into steatohepatitis, cirrhosis, and hepatocellular carcinoma but also achieve a remarkable reduction of T2DM incidence [12–15]. In China, the prevalence of FLD and T2DM is similar in trend. FLD and T2DM are mutually causal, both promoting the onset of cirrhosis, hepatocellular carcinoma, coronary heart disease, and chronic kidney and extrahepatic malignancies such as colorectal cancer [26]. On the one hand, the prevalence of FLD in T2DM is 28-70%. On the other hand, FLD is usually combined with T2DM (22.5%, 95% confidence interval: 17.9-27.9%) [26]. According to the PAF_p_ of FLD in different sex and age groups, male and female populations over 40 years should pay close attention to their health to prevent FLD to reduce T2DM incidence. The findings in the comparison group suggested that male sex, age (≥ 45 years old), and FLD could be considered high-risk populations for T2DM.

Overweight is a common feature as well as an independent risk factor for FLD; it is also a major risk factor of T2DM [1, 25, 27, 28]. In our study, approximately 24.96% of T2DM cases were attributed to overweight, suggesting that maintaining a normal BMI contributes to T2DM prevention. Weight control is also the most effective method to prevent or reverse FLD [25, 29]. Hypertriglyceridaemia and systolic hypertension exist concurrently with FLD [25, 29]. For this reason, decrease in FLD prevalence also lowers the prevalence of overweight, hypertriglyceridaemia, and systolic hypertension; all these together significantly reduce the occurrence of T2DM.

This study has several limitations that should be considered when interpreting our findings. First, the lack of information on other uncontrolled confounding factors, such as social-economic status data, smoking, waist circumference, family history of diabetes and situation of physical activity may lead to an overestimated PAF of FLD. Second, we determined T2DM by FBS. The lack of oral glucose tolerance test and glycosylated haemoglobin may have resulted in the missed diagnosis of some patients with diabetes if they had normal FBS [1, 30–32]. This is a major limitation of the study. Third, our study was based only on patients who underwent a check-up at one centre, which may cause selection bias. Moreover, the shortcomings of a retrospective cohort study, such as the absence of clinical data (circumference, waist circumference, and body fat ratio, among others), may have also limited the analysis.

Despite the limitations, our study had a large population and long-term follow-up. We estimated the PAFs of FLD for T2DM, which are rarely reported. FLD has become the most common chronic liver disease in China, and the prevalence is still increasing [15, 26]. Therefore, we should be vigilant about the occurrence of T2DM caused by FLD. Our study demonstrates that the reduction in the prevalence of FLD is particularly important. Not only FLD itself but also the accompanying overweight, hypertriglyceridaemia, and systolic hypertension could reduce the onset of T2DM; its overall rate may be much higher than 23.11% estimated in our study. Finally, particular attention should be paid to FLD, especially in male populations of 30–40 years old and female population over 40 years.

## Conclusions

Approximately 23.11% of T2DM was attributed to FLD, which was higher than the contribution of other metabolic disorders investigated in this study. Overweight, hypertriglyceridaemia, and systolic hypertension also contributed to T2DM incidence. The PAF_p_ of FLD was especially high in male population of 30–40 years old and the female population over 60 years old. To reduce the economic burden and adverse consequences of T2DM, efforts should be made to reduce the prevalence of FLD.

## Data Availability

The datasets used and/or analysed during the current study available from the corresponding author on reasonable request.
